# *Pleurocinus ostreatus* Polysaccharide Alleviates Cyclophosphamide-Induced Immunosuppression through the Gut Microbiome, Metabolome, and JAK/STAT1 Signaling Pathway

**DOI:** 10.3390/foods13172679

**Published:** 2024-08-25

**Authors:** Daiyao Liu, Abdul Mueed, He Ma, Tianci Wang, Ling Su, Qi Wang

**Affiliations:** 1 Engineering Research Center of Edible and Medicinal Fungi, Ministry of Education, Jilin Agricultural University, Changchun 130118, China; daiyaoliu2023@163.com (D.L.); mahetina@163.com (H.M.); wangtianci0617@163.com (T.W.); suling0648@jlau.edu.cn (L.S.); 2 College of Plant Protection, Jilin Agricultural University, Changchun 130118, China; 3 State Key Laboratory of Food Science and Technology, Nanchang University, Nanchang 330047, China; amueed3723@yahoo.com

**Keywords:** *Pleurocinus ostreatus* polysaccharide, immunomodulatory activity, JAK/STAT1 pathway, gut microbiota, metabolome

## Abstract

This study investigated the structure of *Pleurocinus ostreatus* polysaccharide (POP-1) and its effect on immunocompromised mice induced by cyclophosphamide (CY). Novel POP-1 was α- and β-glucopyranose, its molecular weight was 4.78 × 104 Da, it was mainly composed of glucose (88.9%), and it also contained galactose (2.97%), mannose (5.02%), fucose (0.3%), arabinose (0.21%), ribose (0.04%), galactose acid (0.17%), and glucose acid (1.45%). After POP-1 was administered to immunosuppressed mice, results showed that POP-1 increased the body weight, spleen, and thymus index and enhanced T lymphocyte proliferation in mice. POP-1 up-regulated the expression of CD3^+^, CD4^+^, and CD8^+^ lymphocytes and the ratio of CD4^+^/CD8^+^ in the mouse spleen to increase immunoglobulin (IgM, IgG, and IgA) and secrete cytokines (IL-2, IL-6, TNF-α, and IFN-γ) through activation of the JAK/STAT1 signaling pathway. Moreover, POP-1 remarkably reversed the gut-microbiota dysbiosis in immunosuppressed mice by increasing the abundance of *Muribaculaceae*, *Lactobacillaceae*, *Blautia*, and *Ligilactobacillus* and altered the fecal metabolites by increasing hexahomomethionine, DG(8:0/20:4(5Z, 8Z, 11Z, 14Z)-OH(20)/0:0, 2-((3-aminopyridin-2-yl)methylene)hydrazinecarbothioamide, Ginkgoic acid, and carboxy-ethyl-hydroxychroman, which is closely related to the immunity function. This study indicates that *P. ostreatus* polysaccharide effectively restores immunosuppressive activity and can be a functional ingredient in food and pharmaceutical products.

## 1. Introduction

The immune system plays a pivotal role in maintaining organismal health, as a defense mechanism against exogenous pathogens and internal lesions [1]. Particularly noteworthy is its involvement in various disease states, notably during radiation and chemotherapy of cancer treatment, where immune modulation is essential, as in tumor immunotherapy [2]. Polysaccharides extracted from diverse sources, including plants, animals, and microorganisms, have garnered attention as natural immune enhancers due to their extensive immune activity, biocompatibility, non-toxicity, and degradability [3]. Fungi, renowned for their abundant resources and rapid growth, have emerged as significant sources of polysaccharides with immunomodulatory properties since the initial discovery of mushroom-fruiting body polysaccharides with anticancer effects. Various fungal polysaccharides have been shown to influence immune organ functions, regulate humoral and cellular immunity, promote cytokine production, activate complements, and modulate immune cell signaling pathways [4]. The activation of pattern-recognition receptors by fungal polysaccharides, such as toll-like receptors, induces macrophages to produce proinflammatory cytokines and chemokines, thereby orchestrating immune responses [5]. The JAK/STAT1 pathway is a critical cellular signaling mechanism that mediates various physiological responses, including cell proliferation, differentiation, immune regulation, and apoptosis [6]. Therefore, JAK/STAT1 signaling pathways are prospective treatment targets for immune disorders.

The structural attributes of polysaccharides, including branching, molecular weight, conformation, monosaccharide composition, and glycosidic linkage, significantly influence their immunostimulatory activity. The triple-helix conformation enhances the immunostimulatory activity of *β*-D-glucans in macrophages by facilitating interactions with immune cell surface receptors [7]. Additionally, the molecular weight indirectly influences polysaccharide immunostimulatory properties by impacting solution characteristics and the polysaccharide structure [8,9]. The metabolome refers to the complete set of small-molecule metabolites present within a biological sample; these metabolites include various compounds that are the end products of cellular processes [10]. Its importance lies in its ability to reflect the biochemical activity and metabolic status of cells and tissues [10]. Therefore metabolomic analysis is an important tool for understanding disease mechanisms, discovering biomarkers, and evaluating therapeutic interventions.

*Pleurotus ostreatus* is one of the top edible mushrooms in China. It is renowned not only for its culinary appeal but also for its diverse biological effects. Polysaccharide compounds, considered among the most significant active constituents of *P. ostreatus*, have been extensively studied for their antioxidative, anti-fatigue, and immunomodulatory properties [11]. Recently, it has been reported that *P. ostreatus* polysaccharides improved immune function by increasing T lymphocyte proliferation in mice. However, the effect of *P. ostreatus* polysaccharides on the distribution of T and B cell and lymphocyte subsets, such as CD3^+^, CD4^+^, and CD8^+^ T lymphocytes, in Cy-induced immunosuppressed mice remains unclear. Similarly, different research has been performed on the *P. ostreatus* polysaccharides against immunomodulatory activity; for example, it has been reported that *P. ostreatus* polysaccharides have potential immunomodulatory activity by activating the NF-KB protein in RAW264.7 cells [12]. Similarly, Du et al. reported that *P. ostreatus* polysaccharides can act as antioxidants and enhance the immune function through the activation of immunoglobulin (IgM, IgG, and IgA) and cytokines (IL-2, TNF-α and IFN-γ) [13]. However, *P. ostreatus* polysaccharide’s role in activating the JAK/STAT1 signaling pathway remains unknown. 

Natural sources of polysaccharides exhibit intestinal immunomodulatory activity and act as natural immunomodulators. Therefore, developing possessive polysaccharides with potential intestinal immunomodulatory activity has remained a research focus. *A. auricula* polysaccharides lower pH levels in various parts of the intestinal lumen, promoting commensal microbiota proliferation and increasing the SCFA concentration [14]. Moreover, *A. auricula* polysaccharides have been found to mitigate Cy-induced immunosuppression and modulate Cy-induced intestinal microbiota disruption, as evidenced by a decrease in the *F*/*B* ratio and an increased abundance of *Alloprevotella*, *Blautia*, and *Lactobacillus* [15]. However, the interaction between POP-1 and the gut microbiota remains unknown, particularly regarding its impact on the fecal metabolome, gut microbiota, and JAK/STAT1 pathways. Clarifying these interactions lays the groundwork for exploring POP-1 as a potential probiotic and developing precision nutrition strategies. Therefore, this study aimed to isolate, purify, and characterize POP-1 from *P. ostreatus*, elucidating its molecular weight and composition. Furthermore, we investigated the protective effects of POP-1 on the immune system and gut microbiota in CY-induced immunosuppressed mice to assess its nutritional value. This research contributes to our understanding of the structure-related bioactivities of *P. ostreatus* polysaccharides, paving the way for developing bioactive components for pharmaceuticals and functional foods with immunoregulatory properties.

## 2. Materials and Methods

### 2.1. Extraction of Polysaccharides

The polysaccharide extraction process followed the method described in [16]. Dried fruiting bodies of *P. ostreatus* (500 g) were added to distilled water at a ratio of 1:40 and extracted for 3 h at 70 °C. After filtration, 95% ethanol was added to achieve a final concentration of 70% ethanol, and the mixture was kept at 4 °C overnight. The mixture was centrifuged at 5000 rpm for 15 min to remove the surfactant, and the crude polysaccharide of *P. ostreatus* was collected. The Sevag reagent was added to the crude polysaccharide and stirred for 1 h. The sugar solution was collected after centrifugation at 5000 rpm for 5 min. This process was repeated until no denatured protein was observed after centrifugation, and then, the mixture was freeze-dried. The de-proteinized polysaccharide solution was dialyzed using a membrane with a molecular weight cutoff of 8~12 kDa with running water for 24 h, followed by deionization dialysis for another 24 h. The dialysate was then freeze-dried, and the polysaccharide of *P. ostreatus* (POP) was collected.

### 2.2. Separation and Purification

Polysaccharide was purified according to our previous method [17] with some modification. Polysaccharide (POP, 100 mg) was mixed in 5 mL of ultrapure and dialyzing water for 48 h. After that, the sample was filtered (0.45 μm filter) and then put into a DEAE52 cellulose column, where the sample was eluted using distilled water, as well as NaCl solutions, at diverse mass fractions at a 1 mL/min flow rate for 5 min/tube. Subsequently, the eluent was collected, the sugar content of the eluent in each tube was determined, and the elution curve was plotted. After that, 10 mg of the eluted polysaccharide was mixed with 2 mL of water and eluted on the Sephadex G75 gel column at a 1 mL/3 min controlled flow rate. The sugar content was determined in the eluent at 15 min, and the elution curve was plotted.

### 2.3. Monosaccharide Composition and Molecular Weight Analysis of POP

The POP-1 monosaccharide composition was determined through ion exchange chromatography with the Dionex™ CarboPac™ PA20 column with a 0.5 mL/min flow rate. The injection volume was 5 μL, and the column temperature was 30 °C. The mobile phase A was set to H_2_O, phase B was 200 mM NaOH, and C phase was 200 mM NaOH/500 mM NaAC. The POP-1 molecular weight was determined by using the HPGPC method. The dextran standards with molecular weights of 500, 200, 100, 70, 40, and 10 kDa were used to estimate the MW of POP-1. POP-1 (2 mg) was accurately dissolved in 2 mL of deionized water. Subsequently, 10 μL of sample was injected into a TSK-gel G-4000PWXL chromatographic column (7.8 × 300 mm) at 35 °C, the flow rate was set at 0.6 mL/min, and distilled water was used for a gradient elution. The Agilent 1260 Infinity ELSD evaporative light scattering detector was later adopted to detect the sample, with the retention time being recorded and the molecular weight standard curve being plotted. 

### 2.4. Infrared Spectrum of Polysaccharide

The POP-1 mixture was pressed into a transparent sheet for FT-IR and scanned for the infrared spectra at 4000~400 cm^−1^. Data were averaged from three separate assays.

### 2.5. Animal Experiments

Seventy-five (7-week-old) BALB/c male mice were randomized as five groups (n = 15 each), including the blank control (CON), model (MOD), high-dose H-POP-1 (200 mg/kg), medium-dose M-POP-1 (100 mg/kg), and low-dose L-POP-1 (50 mg/kg) groups. Except for the blank control group, mice in the remaining four groups were given intraperitoneal injections of CY at 80 mg/kg for 5 consecutive days to produce the immunosuppressed model mice. Then, the POP-1 solutions at 200, 100, and 50 mg/kg were administered via gavage to high-, medium-, and low-dose POP-1 groups, respectively, and saline at the same volume was given to the model group and blank group, once a day, for 20 days. Mouse body weight was measured and recorded throughout the experiments. The immune organs, such as the thymus and spleen index, were assessed.

### 2.6. H&E Staining of Spleen

Fresh samples of the spleen and colon were gathered from every mouse group and preserved using 4% paraformaldehyde. Tissue specimens underwent embedding, slicing, and subsequent staining using hematoxylin-eosin (H&E) [18]. Scanning and imaging techniques were used for histopathological observations and analysis.

### 2.7. Distribution of T and B Cells in Mouse Spleen

The spleens of mice treated with ethylenediaminetetraacetic acid antigen repair on frozen and tissue sections cool naturally and decolorize. Slice and incubate in a 0.3% hydrogen oxide solution at room temperature in the dark for 25 min, wash, add 10% normal rabbit serum, and seal at room temperature for 30 min. Add phosphate buffer saline and primary antibody, incubate overnight at 4 °C in a wet box, wash, add secondary antibody, label with horseradish peroxidase, incubate at room temperature for 50 min, and wash. Add diaminobenzidine color solution and Harris re-stain, rinse with tap water, differentiate with 1% hydrochloric acid alcohol, and rinse. The ammonia solution turns blue, rinse again, and follow Section 2.8 to observe the number of positive cells under a microscope.

### 2.8. Lymphocyte Proliferation

The LPS-induced lymphocyte proliferation analysis was followed using the method described by Huo et al. [19] with some modifications. Here, 100 μL of the lymphocyte suspension was seeded in plates; PHA and LPS were added to each plate and incubated for 2 h under saturated humidity conditions at 40 °C. Then, add 20 μL MTT solution (5 mg/mL), incubate for 3 h, centrifuge at 1000 rpm for 10 min, discard the supernatant, and add 150 μL DMSO; fully dissolve the formaldehyde, and detect the absorbance at 590 nm.

### 2.9. Analysis of Lymphocyte Subsets

The blood sample was mixed with an equal volume of PBS solution. Preheat the lymphocyte separation solution and anticoagulant in a water bath, slowly add anticoagulant to the lymphocyte separation solution, centrifuge at 2000 rpm for 20 min, draw the white lymphocyte layer, wash, centrifuge at 4 °C and 1000 rpm for 5 min to remove the supernatant, resuspend the cell culture solution, and adjust the cell count to 1 × 10^6^ cells. Add monoclonal antibodies at ratio of 10:1 to the cell suspension, incubate at 4 °C in the dark for 30 min, wash with PBS, centrifuge at 1000 rpm at 4 °C for 5 min, wash again with PBS, and suspended in 0.5 mL PBS; the counts of CD3^+^, CD4^+^, and CD8^+^ T lymphocytes were measured using a FACSCalibur flow cytometer and expressed as a percentage of the total number of T lymphocytes.

### 2.10. Cytokines and Immunoglobulin Contents

An enzyme-linked immunosorbent assay (ELISA) was conducted to analyze the cytokine content according to the kit instructions, including IL-2(555148), IL-6(555220), TNF-α (555212) and IFN-γ(555138), and immunoglobulins, including IgA(555273), IgG(555247), and IgM(555249), all of which were from BD Biosciences. 

### 2.11. Western Blotting

Cell lysis buffer that contained 1% protease/phosphatase inhibitors was added to lyse splenic tissue homogenates. Then, an ultrasonic cell pulverizer (60 W, 5 s fragmentation, 5 s intervals, 1 min) was applied for fragmentation under cryogenic conditions. The samples were centrifuged for 15 min at 12,000 rpm and 4 °C to collect supernatants for protein quantification. After separation through SDS-PAGE, proteins were electrotransferred onto nitrocellulose (NC) membranes. After that, TBST solution containing 5% defatted milk powder was added to block NC membranes at 4 °C for a 3 h period, followed by a 12 h primary antibody incubation and an additional 40 min incubation using HRP-coupled secondary antibodies at ambient temperature. After washing with TBST for 30 min, protein blots were finally detected using enhanced chemiluminescence. The monoclonal antibodies were from Thermo Fisher Scientific (Waltham, MA, USA), as follows: IL-2, MA5-23785; IL-6, MA5-23797; TNF-α, MA5-23712; IFN-γ, MA5-23683; pJAK1 (Tyr1034/1035), 44-855G; pSTAT1 (Tyr701), 33-3400; GAPDH, MA5-15738; β-actin, MA1-140.

### 2.12. Gut Microbiota Analysis

The TGuide S96 Fecal Genome Kit (4993462, TIANGEN, Beijing, China) was used to isolate DNA from the cecum of each group of mice. The quality and quantity of DNA were evaluated via a concentration assay with an enzyme marker (Gene Company Limited, Beijing, China) and the electrophoresis of PCR amplification products (Fuxin Technology, Foshan, China). Primer 338 F/806 R (5′-ACTCCTACGGGAGGCAGCA-3′/5′-GGACTACHVGGGTWTCTAAT-3′) was used to amplify the V3–V4 region of a bacterial 16 S rRNA gene fragment. The Illumina NovaSeq sequencer was used for standardization and sequencing, employing a double-end sequencing (Paired-End) approach [20].

### 2.13. Fecal Metabolomic Analysis

Untargeted metabolites of feces were identified using HPLC-MS/MS connected with a Q-Exactive Focus (Thermo-fisher Scientific), according to the method [21]. PCA and PLS-DA were performed on the obtained metabolites. Differential metabolites were identified using KEGG (http://www.genome.jp/kegg/, accessed on 2 February 2024), HMDB (https://hmdb.ca/, accessed on 2 February 2024), and MetOrigin (https://metorigin.met-bioinformatics.cn/home/, accessed on 2 February 2024) databases using the criteria of a high confidence (VIP > 1) and significant difference in mean metabolite intensities (*p* < 0.05). Furthermore, Spearman analysis was executed to ascertain the correlation between groups regarding intestinal microbiota and the makeup of diverse metabolites.

### 2.14. Statistical Analysis

SPSS version 26 and GraphPad Prism 8 software were used for all statistical analysis. MetaboAnalyst (http://www.metaboanalyst.ca/, accessed on 2 February 2024) was used to process and analyze the data on intestinal metabolites. A Pearson correlation analysis was used to determine how various bacterial genera correlated with biomarkers of rat characteristics. In several statistical tests, the *p*-value was modified by applying the Benjamini and Hochberg false discovery technique. A result was considered statistically significant if the adjusted *p*-value was less than 0.05.

## 3. Results

### 3.1. Composition of POP-1

**Figure 1:**
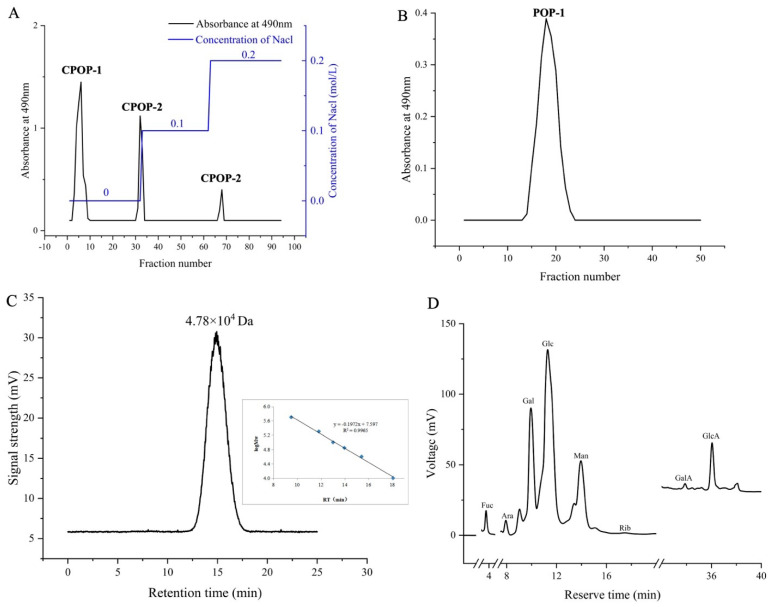
(**A**) Crude polysaccharide profile of *Pleurocinus ostreatus*. (**B**) Elution profile of POP−1 in Sephadex G75 column. (**C**) HPGPC chromatogram of POP−1. (**D**) HPLC profile of monosaccharide standard and POP−1 profile.

**Figure 2:**
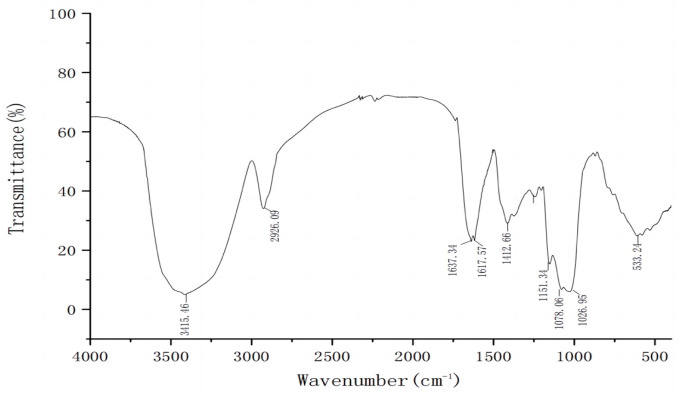
FT-IR spectra of POP-1.

Crude *P. ostreatus* polysaccharide (CPOP) was isolated with a yield of 6.61%. After the fractionation of CPOP using DEAE-52 cellulose chromatography and sequential elution with deionized water, as well as NaCl solutions of varying concentrations, homogeneous polysaccharide CPOP-1, acidic polysaccharide CPOP-2, and CPOP-3 were obtained from the fruiting bodies of *P. ostreatus* (Figure 1A). Due to the high content of CPOP-1, it was chosen for further purification and subjected to Sephadex G75 (2.5 × 40 cm) chromatography. Figure 1B illustrates a single symmetric peak of POP-1 elution obtained from the Sephadex G75 chromatography, indicating homogeneity. The purified POP-1, with a yield of 5.31%, was obtained for further study. The total sugar and uric acid content of POP-1 were 92.73% and 0.39%, respectively. In addition, the hydrographic chromatogram of POP-1 showed a single symmetric peak, indicating that the purity of POP-1 was more than 98.9%, with an average molecular weight of POP-1 being 4.78 × 10^4^ Da (Figure 1C). POP-1 was mainly composed of glucose (88.9%), which dominated, and also contained galactose (2.97%), mannose (5.02%), fucose (0.3%), arabinose (0.21%), ribose (0.04%), galactose acid (0.17%), and glucose acid (1.45%) (Figure 1D). The FT-IR result of POP-1 (Figure 2) showed that a strong absorption peak was detected at 3400 cm^−1^, ascribed to O-H bond stretching vibration. Meanwhile, the asymmetric stretching vibration absorption peak of the hypo methyl C-H2 bond of the polysaccharide at 2920 cm^−1^, the absorption peak of the C=C bond or C=O bond of the polysaccharide at 1637 cm^−1^, the O-H angular shift vibration peak of -COOH at 1412 cm^−1^, or methyl-bending vibration peak in the absence of other functional groups, C-O stretching vibration at 1078 cm^−1^, and absorption peaks at 848 cm^−1^ and 761 cm^−1^ indicated that α-glycosidic bonds existed in POP-1. Moreover, the characteristic absorption peaks for β-glycosidic bonds were detected at around 900 cm^−1^, revealing the existence of β-glycosidic bonds within POP-1. Therefore, it confirms that POP-1 was α- and β-glucopyranose.

### 3.2. Effect of POP-1 on Body Weight and Immune Organ Index of Mice

**Figure 3:**
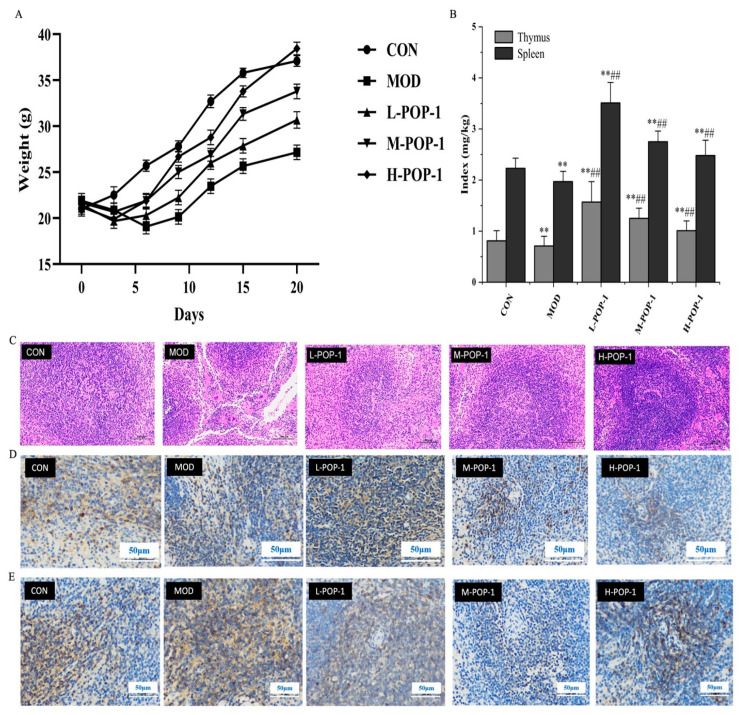
(**A**) Effect of POP-1 on mouse body weight. (**B**) Effect of POP-1 on immune organ index of mice. H&E staining and distribution of CD3^+^ T and CD4^+^ T lymphocytes in the spleen. (**C**) H&E staining. (**D**) CD3^+^ T lymphocytes. (**E**) CD4^+^ T lymphocytes. Compared with CON, ** (*p* < 0.01). Compared with the MOD group, ## (*p* < 0.01). Data are presented with n = 3.

The thymus and spleen indices are two vital immune organs that can measure immunological functions. Health problems are also tightly correlated with body weight; intraperitoneal injections of cyclophosphamide have various effects on the growth of mice [22]. Figure 3A shows that CY reduces the body weight of the mice. Compared to the MOD group, other groups containing different doses of POP-1 increased the body weight of mice. Our results show a significant difference (*p* < 0.05) between the MOD and H-POP-1 groups, while no significant difference was found between the L-POP-1 and MOD groups.

Similarly, Figure 3B shows that the administration of CY affected the mouse thymus and spleen index. The spleen and thymus index in the MOD group decreased compared to the CON group. However, compared to the MOD group, there were highly significant differences in the spleen and thymus index of the M-POP-1 and H-POP-1 groups (*p* < 0.01). Therefore, we speculated that POP-1 protects the peripheral and immune organs in immunosuppressive mice.

### 3.3. HE Staining of Spleen and Splenic Lymphocytes

The spleen is the largest peripheral lymphoid organ in the human body and the primary site for immune responses. Therefore, through H&E staining, it was found that the spleen of the CON group mice developed well, with a rich and developed distribution of red and white pulp (Figure 3C). The spleen morphology of the H-POP-1 group was similar to that of the CON group, with no significant difference. Compared with the CON group, the spleen cells of the MOD-group mice were loosely arranged, with a considerable reduction in the number of red and white pulps and blurred boundaries. The lymphoid sheaths were atrophied, the number of splenic corpuscles was reduced, and large cracks appeared at the splenic cord. However, there was no significant difference between the L-POP-1 and MOD groups, while a significant difference was found between the H-POP-1 and MOD groups (*p* < 0.05). Therefore, our results indicated that POP-1 restores the spleen tissue morphology and immune function dose-dependently (Figure 3C).

Figure 3D showed that the MOD group had a significantly reduced number of CD3^+^ positive cells, fewer red pulps, more giant splenic cord fissures, and a loose cell structure. The number of CD3^+^ positive cells increased dramatically after treating immunosuppressive mice with the H-POP-1, and the positive cells mostly gathered around the lymphatic sheath. The morphology of the spleen tissue was intact, with a regular distribution, and no splenic cord cracks were observed, similar to that of the CON group. Our results showed that medium and low-dose POP-1 groups can also improve the distribution of CD3^+^ T lymphocytes in the spleen.

Figure 3E shows the distribution of CD4^+^ T lymphocytes in the spleen. Compared to the CON group, the MOD group only exhibited a minimal number of lymphatic sheaths, and the number of CD4^+^ positive cells was significantly reduced. CD4^+^ positive cells were significantly increased when the immunosuppressive mice were treated with high-dose POP-1. The positive cells in the H-POP-1 group were denser than those in the MOD group. However, the CD4^+^ T lymphocytes increased in the M-POP-1 and L-POP-1 groups compared to the MOD group. Therefore, our results showed that POP-1 restores the CD3^+^ T and CD4^+^ T lymphocytes in the spleen of immunosuppressive mice treated with CY.

### 3.4. Splenic Lymphocyte Proliferation

Splenic lymphocyte proliferation is directly and indirectly associated with irregularities in T and B lymphocytes [23]. Therefore, in this study, we induced proliferation in T and B splenic lymphocyte cells using PHA and LPS (Table 1). Compared with the model group, there was a significant difference in lymphocyte proliferation between the medium- and high-dose treatment groups (*p* < 0.01). In contrast, the low-dose treatment group showed a significant difference in lymphocyte proliferation (*p* < 0.05). Compared with the CON group, the high-dose treatment group showed potentially significant differences in T and B lymphocyte proliferation. In contrast, the medium- and low-dose treatment groups showed significant differences in lymphocyte proliferation (*p* < 0.05). The above results indicate that POP-1 promotes the proliferation of T and B lymphocytes in the blood of immunosuppressed mice.

### 3.5. Spleen T Lymphocyte Subsets

Different cell surface markers are involved in the regulation of the immune function of T lymphocytes, while CD3^+^ is the significant marker of T cells. CD4^+^ and CD8^+^ are mainly found in mature T cells and act as T lymphocyte helper and effector cells in the innate immune response [24]. The percentage of CD3^+^, CD4^+^, and CD8^+^ T lymphocytes and the ratio of CD4^+^/CD8^+^ in the MOD group were reduced (*p* < 0.01) compared to the CON group. The percentage of CD3^+^, CD4^+^, and CD8^+^ lymphocytes increased in immunosuppressive mice after being treated with different doses of POP-1 (Table 2). The high-dose POP-1 group indicated significant differences in the number of CD3^+^ positive cells compared to the CON group (*p* < 0.05). Compared with the MOD group, the percentage of CD3^+^, CD4^+^, and CD8^+^ lymphocytes significantly increased after the administration of POP-1, except for the percentage of CD8^+^ and the CD4^+^/CD8^+^ ratio in the L-POP-1 group. In contrast, the percentage of CD3^+^, CD4^+^, and CD8^+^ lymphocytes and the CD4^+^/CD8^+^ ratio in the other dose groups showed particularly significant differences (*p* < 0.01).

### 3.6. Effect of POP-1 on Immunoglobulin and Cytokines

**Figure 4:**
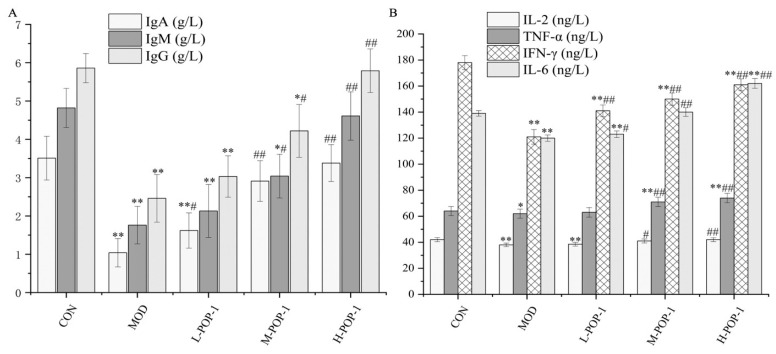
(**A**) Effect of POP-1 on serum immunoglobulin in immunosuppressive mice. (**B**) Effect of POP-1 on cytokines in serum of immunosuppressive mice. Compared with CON, * (*p* < 0.05), ** (*p* < 0.01). Compared with the MOD group, # (*p* < 0.05), ## (*p* < 0.01). Data are presented with n = 3.

To further explore the immunomodulatory activity of POP-1, serum levels of immunoglobulins and cytokines were measured (Figure 4). Figure 4A indicates that immunoglobulin levels were decreased in the MOD group compared to the CON group (*p* < 0.05). In contrast to the MOD group, serum IgM, IgG, and IgA secretion levels were effectively increased after mice were treated with POP-1 (*p* < 0.01). Moreover, the serum levels of IgM, IgG, and IgA also increased in mice treated with the medium (*p* < 0.01) and low dose (*p* < 0.05) of POP-1. These results suggest that POP-1 can effectively promote the production of immunoglobulins in immunosuppressed mice and restore the humoral immune function of the body.

Figure 4B demonstrates that cytokine levels decreased in mice treated with CY. Compared with the CON group, the MOD group showed a significant decrease in IL-2, IL-6, TNF-α, and IFN-γ levels (*p* < 0.05). However, IL-2, IL-6, TNF-α, and IFN-γ levels increased in immunosuppressed mice treated with different doses of POP-1. Specifically, the H-POP-1 group and M-POP-1 group exhibited highly significant differences compared to the MOD group (*p* < 0.05). These results suggest that POP-1 can promote the release of immune factors and increase serum cytokine secretion in mice dose-dependently.

### 3.7. Effect of POP-1 on JAK/STAT Pathway

**Figure 5:**
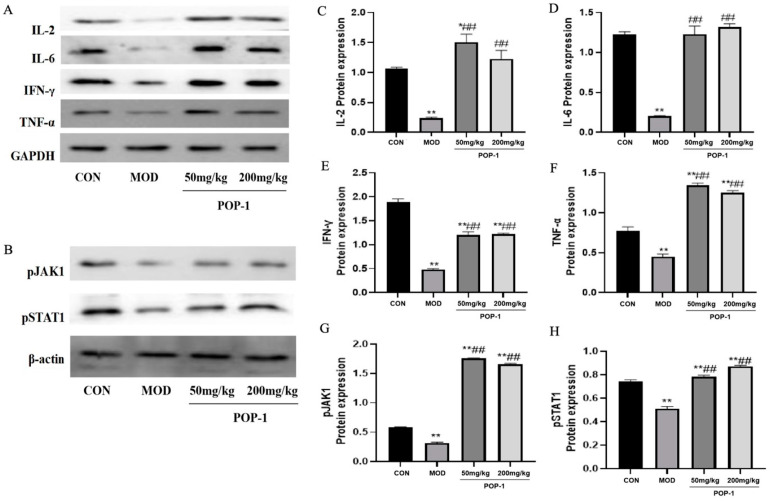
Effect of POP-1 on JAK/STAT1 signaling pathway in the spleens of immunosuppressive mice. (**A**,**B**) Protein level was analyzed via Western blotting. (**C**–**H**) Protein expression level. Compared with CON group, * (*p* < 0.05), ** (*p* < 0.01). Compared with the MOD group, ## (*p* < 0.01). Data are presented with n = 3.

The JAK/STAT1 signaling pathway is crucial in regulating the activation of critical immune mediators, such as IL-2, IL-6, INF-γ, and TNF-α [25]. To investigate this pathway, we assessed the phosphorylation levels of these four factors, along with STAT1 and JAK, using Western blotting (Figure 5). In immunosuppressed mice treated with POP-1, the protein levels of IL-2, IL-6, TNF-α, and INF-γ were up-regulated (Figure 5A). Conversely, compared to the CON group, the phosphorylation levels of STAT1 protein and JAK2 protein were reduced in the MOD group. However, treatment with POP-1 led to an up-regulation of the phosphorylation levels of STAT1 protein and JAK2 protein compared to the MOD group (Figure 5B). These findings suggest that POP-1 may exert its immunomodulatory effects in cyclophosphamide-induced mice by modulating the JAK/STAT1 pathway.

### 3.8. Effect of POP-1 on Gut Microbiota

**Figure 6:**
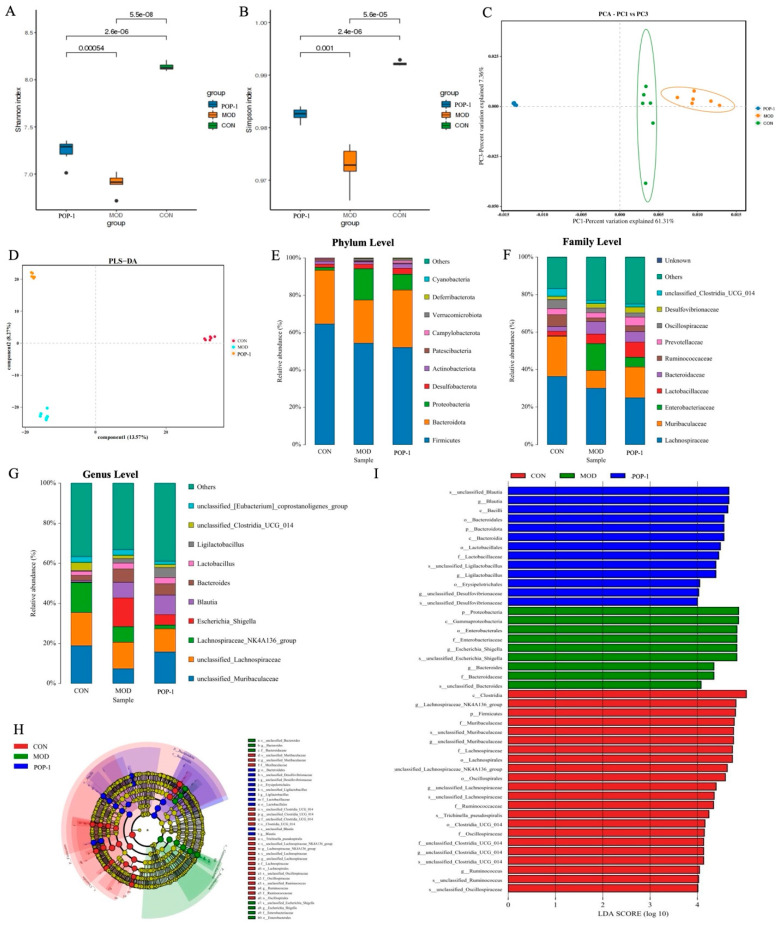
Effect of POP−1 on gut microbiota in immunosuppressive mice. α-Diversity analysis (**A**,**B**); *β*-diversity analysis (**C**,**D**); (**E**) ratio of microbial abundance at the phylum level; (**F**) ratio of microbial abundance at the family level; (**G**) ratio of microbial abundance at the genus level; (**H**) cladogram of LEFSe analysis; (**I**) LEFSe analysis among control, MOD, and POP−1 groups; lowercase letters indicate a significant difference, *p* < 0.05, based on ANOVA followed by Duncan’s test. Data are presented with n = 6.

According to the above results, H-POP-1 was selected to conduct 16S rRNA analysis to investigate gut microbiota’s richness, diversity, and composition in the cecum of immunosuppressed mice following treatment with POP-1. Alpha-diversity metrics, including Shannon and Simpson indices (shown in Figure 6A,B), revealed a significant decrease in all four indicators in the MOD group compared to the CON group. Notably, POP-1 administration reversed this trend, considerably increasing the Simpson and Shannon indices. Furthermore, beta-diversity parameters were assessed using PCoA and PLS-DA (Figure 6C,D), indicating substantial variation in the species composition between samples. These findings suggest that cyclophosphamide-induced immunosuppression reduces the gut microbiota number and diversity, with POP-1 intervention potentially playing a crucial role in restoring the microbial community diversity and richness.

Moreover, taxonomic analysis at the phylum, family, and genus levels revealed insights into the bacterial composition (Figure 6E–G). At the phylum level, *Proteobacteria*, *Bacteroidota*, and *Firmicutes* were predominant in the mice. Following POP-1 treatment, there was a decrease in the abundance of Proteobacteria and *Firmicutes/Bacteroidota* compared to the MOD group. At the family level, POP-1 administration led to a notable reduction in Enterobacteriaceae and Bacteroidaceae abundance, while the relative abundance of *Muribaculaceae* and *Lactobacillaceae* increased. At the genus level, POP-1 intervention in CY-treated mice altered the relative abundances of microbiota, including *unclassified_Muribaculaceae*, *Blautia*, and Ligilactobacillus.

Furthermore, LEfSe analysis was employed to further investigate the impact of POP-1 on functional alterations induced by CY in the gut microbiota (Figure 6H). Using a linear discriminant analysis (LDA) effect size (LEfSe) score threshold >4, characteristic taxonomic abundance (n = 7) was identified to elucidate the crucial taxonomic shifts in gut bacterial diversity and communities (Figure 6I). Our results revealed that the pathogenic family *Bacteriodeaceae* and the genus *Escherichia_Shigella* were predominant in the MOD group. Conversely, significant differences in family- and genus-level species were observed in the POP-1 group, including *Lactobacillus*, *Blautia*, and *Ligilactobacillus*.

### 3.9. Effect of POP-1 on Metabolites of Fecal Samples

**Figure 7:**
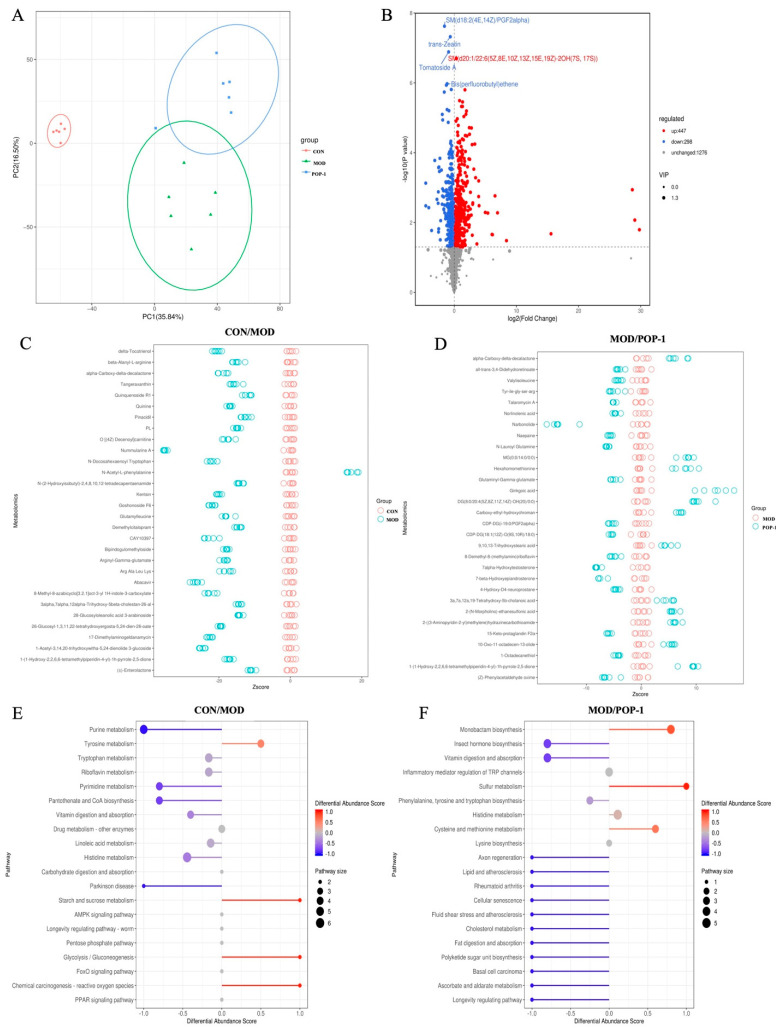
POP−1 supplementation alters the fecal metabolite level in CY-treated immunosuppressed mice. (**A**) Score plot of the PCA model, (**B**) volcano plot, (**C**,**D**) Z-score test, and (**E**,**F**) metabolomic pathway enrichment analysis. Data are presented with n = 6.

The non-targeted metabolomics approach was employed in this study to explore the variation in fecal metabolites among the three groups (CON, MOD, and POP-1). The PCA and PLS-DA model score scatter plots revealed distinct differences among these groups, indicating notable metabolic alterations induced by CY and further modulated by POP-1 treatment (Figure 7A). Further, a volcano plot analysis identified 757 potential biomarkers differently expressed between the MOD and POP-1 groups, with 447 significantly up-regulated and 298 significantly down-regulated metabolites responding to POP-1 intervention (Figure 7B). The reliability of these metabolites’ Z-scores confirms the volcano plots’ reliability (Figure 7C,D). Moreover, the impact of CY on the metabolic pathway of mice was primarily dominated by six metabolic pathways, including purine metabolism, tyrosine metabolism, tryptophan metabolism, pyrimidine metabolism, pantothenic acid and CoA metabolism, and histidine metabolism (Figure 7E,F). Notably, the tyrosine metabolism pathway exhibited an up-regulated trend, while purine metabolism, tryptophan metabolism, pyrimidine metabolism, pantothenic acid and CoA metabolism, and histidine metabolism displayed down-regulated trends. Following POP-1 intervention, significant alterations were observed in metabolic pathways, such as monochitosan metabolism, vitamin digestion and absorption, phenylalanine, tyrosine and tryptophan biosynthesis, histidine metabolism, and cysteine and methionine metabolism. Particularly noteworthy was POP-1’s ability to reverse the down-regulation of histidine metabolism in CY-induced immunosuppressed mice and modulate the biosynthesis level of tryptophan. These findings underscore the potential of POP-1 to profoundly impact metabolic pathways associated with immune functions, highlighting its therapeutic potential in mitigating immunosuppressive effects.

### 3.10. Correlation of Biomarkers with Gut Microbiota and Metabolites

**Figure 8:**
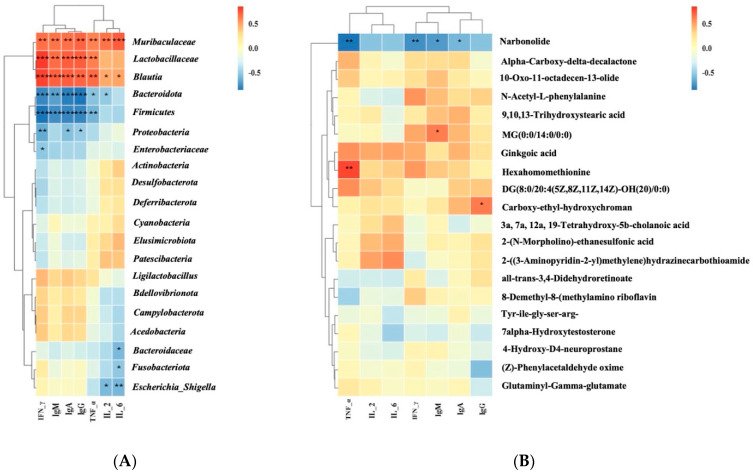
Correlation among differential gut metabolites, gut microbiota, and biomarkers. (**A**) Correlation between differential gut microbiota and biomarkers related to immunosuppression. (**B**) Correlation between the differential gut metabolites and biomarkers related to immunosuppression; a Spearman analysis was used for the matrix (* *p* < 0.05, ** *p* < 0.01, *** *p* < 0.001).

The underlying mechanism of the effects of POP-1 on immunosuppression was further explored by identifying the correlation of gut microbiota and fecal metabolites with markers of immunosuppression. The relative abundance of *Muribaculaceae*, *Lactobacillaceae*, *Blautia*, and *Ligilactobacillus* was positively correlated with IgM, IgG, IgA, IL-2, IL-6, TNF-α, and IFN-γ. In contrast, *Bacteroidota*, *Firmicutes*, *Proteobacteria*, and *Bacteroidaceae* were negatively correlated with IgM, IgG, IgA, IL-2, IL-6, TNF-α, and IFN-γ (Figure 8A). Interestingly, *Ligilactobacillus* was positively correlated with IgM, IgG, IgA, TNF-α, and IFN-γ while negatively correlated with IL-2 and IL-6. Furthermore, the correlation between the primary differential metabolites and immunosuppression markers was analyzed (Figure 8B). The contents of Ginkgoic acid, hexahomomethionine, and carboxy-ethyl-hydroxychroman were positively correlated with IgM, IgG, IgA, IL-2, IL-6, TNF-α, and IFN-γ. In contrast, Narbonolide and 7alpha-hydroxytestosterone were negatively correlated with IgM, IgG, IgA, IL-2, IL-6, TNF-α, and IFN-γ. These outcomes indicate that POP-1 supplementation altered the gut microbiota and produced different metabolites vital in regulating immunosuppression disease.

## 4. Discussion

Polysaccharides constitute a class of biomolecules widely distributed in plants, animals, and microorganisms, exhibiting similarities to nucleic acids and proteins. Their biological activities are intricately linked to their structural attributes, encompassing their content, purity, molecular weight, monosaccharide composition, glycosidic bond type, and sugar chain conformation. In this study, we extracted novel polysaccharides (POP-1) from the fruiting bodies of *P. ostreatus*, yielding 5.31%, with total sugar and uronic acid contents of 92.73% and 0.39%, respectively. It has been reported that linear polysaccharides of β (1→3) glucan exhibit significant immune-enhancing and antitumor activities [26]. Similarly, polysaccharides extracted from *P. ostreatus* with a molecular weight of 3.034 × 10^3^ Da, composed of Man, Glu, Gal, and Xyl, demonstrated potential neuroprotective activity by mitigating oxidative damage in PC12 cells [27]. In our study, the purified POP-1 from *P. ostreatus* comprised α-type and β-type glucopyranose predominantly composed of Glc and Gal, Man, Fuc, Fru, and Ara, with a molecular weight of 4.78×10^4^ Da. This confirms that POP-1 was α- and β-glucopyranose based on FT-IR. The biological activity of polysaccharides may vary depending partly on their molecular weight (Mw) and chemical structure [28]. Soluble low-molecular-weight β-glucans strongly inhibit reactive oxygen species production, acting as antagonists of Dectin-1-mediated signaling [29]. A polysaccharide with a molecular weight of 12.30 kDa has been isolated from Puerariae radix, in which the main linker modes are 1,4-α-D-Glc and 1,3,6-α-D-Glc. This polysaccharide displayed immunomodulatory activity [30]. Moreover, the monosaccharide composition, especially uronic acid and glycosidic linkages in polysaccharides, also influences their bioactivities [22]. A recent report has also demonstrated that natural polysaccharides protect the liver. For example, a novel polysaccharide (Mw = 21.70 kDa) with a backbone consisting of →4)-α-Galp-(1→4)-α-Galp-(1→2)-α-Manp-(1→4)-α-Galp-(1→2)-α-Manp-(1→4)-α-Galp-(1→4)-α-Galp-(1→2)-α-Manp-(1→4)-α-Galp-(1→2)-α-Manp-(1→ was isolated from Coriolus versicolor, which was reported to exhibit a significant effect on protecting the liver by reducing oxidative stress and regulating autoimmunity [31]. Polysaccharide POP-1 from *P. ostreatus*, isolated and purified in this study, had a relatively high molecular weight of approximately 4.78 × 10^4^ Da. Therefore, we speculate that POP-1 would exhibit greater immunomodulatory activity.

The immune organs serve as pivotal sites for immunoreactivity, orchestrating protective responses to eliminate harmful stimuli. The thymus and spleen indexes are essential biomarkers of the immune system, with the thymus facilitating T cell differentiation and the regulation of immunocytes [32], and the spleen is the site for T and B cell colonization and immunoreactivity [33]. *P. ostreatus* polysaccharide has shown promise as a protective agent against CY-induced injury in immune organs [13]. Consistent with these findings, POP-1 significantly increased thymus and spleen indexes in immunosuppressed mice. Similarly, polysaccharides have been shown to modulate dendritic cell maturation and cytokine production to elicit immunological responses [34]. Dendritic cells express pattern recognition receptors, such as C-type lectins and TLRs. Polysaccharides can stimulate them through the TLR4-mediated signaling pathway, thereby enhancing dendritic cell maturation and immune responses [34]. Macromolecular polysaccharides from G. frondosa and C. paliurus have demonstrated immunomodulatory effects by increasing the percentage of CD4^+^ and CD8^+^ T cells and enhancing splenocyte proliferation, respectively [24,35]. Our results indicate that POP-1 restores immune functions by enhancing T and B lymphocyte proliferation, as well as increasing the percentages of CD3^+^, CD4^+^, and CD8^+^ lymphocytes and the CD4^+^/CD8^+^ ratio in CY-induced immunosuppressed mice. CD4^+^ T cells can differentiate into Th1 and Th2 cell subtypes based on cytokine production, with Th1 cells secreting positive regulatory factors, such as IFN-γ, TNF-α, and IL-2 [36]. In contrast, IL-4 secreted by Th2 cells acts as a negative regulatory factor [37]. In addition, IFN-γ IL-4 and IL-4 are a pair of critical immune cytokines that antagonize each other [38]. In addition, cytokines are crucial for regulating immune responses directly or indirectly. IL-2 can enhance the activity of NK cells, cytotoxic T cells, monocytes, and macrophages and promote the proliferation of B lymphocytes and antibody secretion [39]. IL-7 and IL-2 are recognized as pro-survival homeostatic cytokines that are necessary for the growth and upkeep of T and B lymphocytes in lymphoid tissue [40]. However, IL-6 cytokines facilitate the production of IgA, and IFN-γ production increases the level of IgG and IgM [41,42]. Polysaccharides increased the secretion of IgA expression [43]. Song et al. reported that *Glycyrrhiza* polysaccharide with a Mw of 6.5 kDa exhibited potential immunomodulatory activity by increasing immunoglobulin cytokines (IgG and IgM) [44]. Our results demonstrate that POP-1 up-regulates serum immune factors, such as IL-2, IL-6, IFN-γ, and TNF-α, and significantly increases serum immunoglobulin production, including IgM, IgG, and IgA, in immunosuppressed mice, suggesting that POP-1 promotes T lymphocyte differentiation into Th1 cells, which in turn induce B lymphocyte differentiation and maturation, resulting in enhanced cellular and humoral immunity in immunosuppressed mice. Thus, we hypothesize that POP1 protects against immunosuppression in mice by up-regulating serum immune factors, such as IL-2, IL-6, IFN-γ, and TNF-α, and significantly increasing serum immunoglobulin production, including IgM, IgG, and IgA, thereby enhancing both cellular and humoral immunity.

Furthermore, the immunomodulatory effects of POP-1 are closely associated with cytokines, such as IL-2, IL-6, IFN-γ, and TNF-α, in immunosuppressed mice. These four cytokines bind to their respective receptors, activating and transmitting the signals to JAK [25]. JAK phosphorylation is associated with the regulation of cytokines. It recruits STAT to shift to the vicinity of the receptors, eventually controlling the transcription of cellular genes and thus affecting the biological functions of cells [25]. Shan et al. [45] reported that *P. cyrtonema* polysaccharide activates the JAK/STAT1 pathway by reducing the activity of JAK1 in cells and elevating p-STAT3, resulting in the regulation of immunosuppressive activity. IL-2, IL-6, INF-γ, and TNF-α levels and the phosphorylation levels of STAT1 and JAK were down-regulated in mice under the effect of CY.

In contrast, the phosphorylation levels of these four factors increased in mice administered POP-1. Additionally, POP-1 up-regulated STAT1 and JAK1 phosphorylation levels, consistent with the improved blood routine indices of hematopoietic and inflammatory cytokines IL-6 and TNF-α. Thus, POP-1 may exert its immunomodulatory effect in CY-induced mice by regulating the JAK/STAT1 pathway. Our results are consistent with Wang et al. [46], who reported that *P. sibricum* polysaccharide decreased JAK1 and STAT1 protein levels in mice with blood deficiency syndrome. Therefore, we speculate that the immune-regulatory function of POP-1 on CY-induced immunosuppression may be achieved by regulating the JAK/STAT1 signaling pathway.

Host homeostasis and health depend heavily on the gut microbiota, and CY-induced immunosuppression often leads to intestinal dysbiosis characterized by decreased bacterial population diversity and increased potential pathogen numbers [39]. POP-1 reduced the ratio of *F/B* and down-regulated *Proteobacteria* abundance at the phylum level. Xu et al. also reported that Phyllanthus emblica polysaccharide decreased *F/B* values in immunosuppressive mice [47]. Additionally, Proteobacteria has also been linked to intestinal inflammation because many of the taxa associated with this species are opportunistic or pathobionts, and changes in *Proteobacteria* abundance have been regarded as a marker of gut microbial dysbiosis [48]. Similarly, our results showed that POP-1 resists the immunosuppressive effects of CY mainly by increasing the abundance of beneficial bacteria (*Muribaculaceae*, *Lactobacillaceae*, *Blautia*, and *Ligilactobacillus*) and reducing the abundance of harmful bacteria (*Enterobacteriaceae* and *Bacteroidaceae*). It has been reported that Enterobacteriaceae may cause inflammation disease [49], and the elevation of *Bacteroidaceae* promotes benzene-induced inflammation and hematopoietic damage [50]. A recent research report indicated that Gegen Qinlian decoction ameliorates murine colitis by inhibiting the expansion of *Enterobacteriaceae* [51]. *Muribaculaceae* and Lactobacillus were significantly associated with immunoregulatory functions.

Meanwhile, in the report on ginger polysaccharide protection against CY-induced immunosuppression, *Muribaculaceae* and *Lactobacillus* were enriched in the polysaccharide treatment group, which is consistent with the changing trend in this study [52]. According to these research reports, our result showed that the predominant bacteria in the POP-1-treated mice were *Lactobacillus*, *Blautia*, and *Ligilactobacillus*. In addition, *Ligilactobacillus* can be antibacterial and immune and enhance the intestinal barrier [53]. Moreover, bacteria belonging to the *Blautia* genera are vital for maintaining gut health by producing organic acids and vitamins [54]. Several articles reported that the abundance of *Blautia* was decreased in the related studies on CY-induced immunosuppression, and Blautia was negatively correlated with inflammatory factors [55]. Therefore, we speculate that *Blautia* may participate in the immunomodulatory effects by regulating the inflammatory response. At the same time, POP-1 increases the functional metabolites in the gut, resulting in KEGG pathway enrichment analysis, which shows that the differentially expressed metabolic pathways following POP-1 treatment involved lysine degradation, tryptophan metabolism, carbapenem biosynthesis, and alpha-linolenic acid metabolism. In summary, these results indicate that POP-1 alleviates CY-induced immunosuppression by enhancing the diversity of gut microbiota and restoring the relative abundance of probiotics while probably inhibiting the growth of pathogens. However, further study must explore the mechanism underlying the effects of gut microbes, such as *Lactobacillus*, *Blautia*, and *Ligilactobacillus*, on CY-induced immunosuppression. 

## 5. Conclusions

This study obtained a novel water-soluble *P. ostreatus* polysaccharide (POP-1). The main monosaccharide composition of POP-1 was glucose, galactose, and mannose with a molecular weight of 4.78 × 10^4^ Da, and its composition was α- and β-glucopyranose. POP-1 showed potential immunomodulatory activity in immunosuppressed mice by promoting lymphocyte proliferation, increasing serum immunoglobulins, such as IgM, IgG, and IgA, and enhancing cytokines secreted, such as IL-2, IL-6, IFN-γ, and TNF-α, by activating the JAK/STAT1 signaling pathway. However, POP-1 restored the gut microbiota dysfunction and increased the fecal metabolites, which is closely related to the immunomodulatory function. Future research endeavors should delve deeper into elucidating the precise mechanisms underlying the immunomodulatory effects of POP-1, particularly its interactions with the gut microbiota and host immune cells. Furthermore, clinical studies are warranted to validate the therapeutic potential of POP-1 in managing immunosuppression-related disorders in humans. Overall, our findings lay the groundwork for harnessing the immunotherapeutic properties of polysaccharides to develop novel interventions targeting immune dysregulation and enhancing host defense mechanisms.

## Figures and Tables

**Table 1:** Effect of POP-1 on the value-added function of T and B lymphocytes.

Groups	T Lymphocyte	B Lymphocyte
CON	0.532 ± 0.049	0.519 ± 0.056
MOD	0.448 ± 0.053	0.456 ± 0.044
POP-1 (50 mg/kg)	0.487 ± 0.062 ^bc^	0.473 ± 0.058 ^ac^
POP-1 (100 mg/kg)	0.531 ± 0.057 ^d^	0.612 ± 0.045 ^ad^
POP-1 (200 mg/kg)	0.634 ± 0.074 ^ad^	0.748 ± 0.067 ^bd^

Note: compared with CON: ^a^ (*p* < 0.05), ^b^ (*p* < 0.01); compared with MOD: ^c^ (*p* < 0.05), ^d^ (*p* < 0.01).

**Table 2:** Effect of POP-1 on lymphocyte subsets in immunosuppressive mice.

Groups	CD3^+^	CD4^+^	CD8^+^	CD4^+^/CD8^+^
CON	46.02 ± 3.25	28.06 ± 2.20	15.71 ± 1.33	1.79 ± 0.17
MOD	25.89 ± 4.67 b	19.67 ± 2.45 b	13.82 ± 1.54 b	1.42 ± 0.16 b
POP-1 (50 mg/kg)	28.75 ± 2.36 bd	21.92 ± 2.43 bd	14.06 ± 1.83 bc	1.55 ± 0.13 bc
POP-1 (100 mg/kg)	32.09 ± 1.24 ad	24.81 ± 2.69 ad	14.95 ± 1.59 ad	1.66 ± 0.17 bd
POP-1 (200 mg/kg)	38.83 ± 2.63 ad	27.58 ± 2.84 d	15.39 ± 1.31 d	1.79 ± 0.22 d

Note: compared with CON: a (*p* < 0.05), b (*p* < 0.01); compared with MOD: c (*p* < 0.05), d (*p* < 0.01).

## Data Availability

The original contributions presented in the study are included in the article, further inquiries can be directed to the corresponding author.
